# Congenital Cytomegalovirus Infection in Fetal Demise: A Retrospective Cohort Study

**DOI:** 10.3390/v18080881

**Published:** 2026-08-12

**Authors:** Ryan H. Rochat, Gail J. Demmler-Harrison, Magdalena Sanz Cortes, John Diaz-Decaro, Colin Kunzweiler

**Affiliations:** 1 Department of Pediatrics, Baylor College of Medicine, Texas Children’s Hospital, Houston, TX 77030, USA; gdemmler@bcm.edu; 2 Department of Obstetrics and Gynecology, Maternal Fetal Medicine, Baylor College of Medicine, Texas Children’s Hospital, Houston, TX 77030, USA; 3 Causal Inference & AI in Real World Evidence, Black Swan Causal Labs, Los Angeles, CA 91306, USA; jdiazdecaro@gmail.com; 4 ModernaTX, Inc., Cambridge, MA 02142, USA; colin.kunzweiler@modernatx.com

**Keywords:** congenital cytomegalovirus infection, stillbirth, fetal death, cytomegalovirus, pregnancy complications, infectious

## Abstract

Background: CMV is the most common congenital infection worldwide, affecting 0.2–6% of live births and causing significant morbidity and mortality. Although most infections are asymptomatic, severe cases can lead to fetal demise. Objective: To quantify and characterize confirmed CMV infection among fetal demises in a large pediatric hospital in the US from 2013 to 2024. Methods: A retrospective electronic health record (EHR) review of pregnancies with CMV testing, delivery summaries, and confirmed fetal CMV infection via tissue or autopsy linkage. Results: During the review period, 61,973 pregnancies were recorded, with 1173 resulting in fetal demise. Among 1670 pregnancies tested for CMV, 859 had positive testing (e.g., CMV IgG, CMV IgM, CMV DNA), including 96 demises, 94 (11%) of which we had complete delivery records. Four (4%) of these fetal demises were directly attributable to CMV based on positive CMV testing from amniocentesis, fetal tissue, or placenta. Most demises occurred in the second or third trimester (mean gestational age 28.4 ± 6.6 weeks), while the four CMV-attributable cases averaged 23.7 ± 3.7 weeks. Among 93 fetal demise pregnancies in which maternal CMV IgM testing was performed, 9 (9.7%) had a positive CMV IgM result. All confirmed CMV-attributable fetal demises occurred among these CMV IgM-positive pregnancies. Conclusion: These findings underscore CMV’s role in perinatal mortality and highlight the need for improved screening and prevention strategies.

## 1. Introduction

Cytomegalovirus (CMV) is the most common congenital infection worldwide and a leading cause of neurodevelopmental impairment, vision loss, and sensorineural hearing loss [1]. Although most maternal infections during pregnancy are asymptomatic, primary infection carries a transmission risk of up to 40%, with recurrent infections in pregnancy also contributing to congenital infection and disease [2]. Globally, the birth prevalence of congenital CMV (cCMV) infection ranges from 0.18% to 6.1%, with higher rates in regions of elevated maternal seroprevalence [3]. In the United States alone, an estimated 14,000 to 21,000 infants are born with cCMV infection annually [4], and approximately 3000 to 4000 of these children will experience long-term sequelae such as developmental disabilities, hearing loss, or vision impairment [5].

Severe cCMV disease can manifest as intrauterine growth restriction, hydrops fetalis, hepatosplenomegaly, thrombocytopenia, and central nervous system abnormalities, including microcephaly, ventriculomegaly, and intracranial calcifications [6]. CMV is frequently detected in placental and fetal specimens from stillbirths and spontaneous abortions, with a median positivity rate of approximately 7% [7,8,9]; however, most studies rely on histopathologic detection without standardized criteria for causal attribution, limiting inference about CMV as a proximate cause of demise [10].

Where prior studies have demonstrated frequent detection of CMV in placental and fetal tissues after stillbirth, the extent to which CMV can be definitively attributed as the proximate cause of fetal demise in contemporary clinical practice remains unclear. This study quantifies and characterizes confirmed congenital CMV infection among cases of fetal demise. By linking maternal records to fetal tissue or autopsy findings, we provide robust evidence of CMV’s contribution to perinatal mortality.

## 2. Methods

We conducted a retrospective cohort study at Texas Children’s Hospital, reviewing pregnancies documented in the electronic health record (EHR) that included a delivery summary and resulted in fetal demise. The study period spanned pregnancies from 2013 to 2024. Our EHR allows us to track diagnoses assigned during pregnancy in addition to tracking any lab components performed during these pregnancies. To identify pregnancies in which CMV may have been clinically relevant, EHR searches included any documented positive CMV laboratory result, including CMV IgG, CMV IgM, and CMV DNA. Because laboratory ordering practices varied over the study period, CMV DNA searches were intentionally broad and included multiple specimen types and order names. These searches were used solely for case identification and were followed by detailed manual chart review to verify clinical context and classify cases.

The causes of fetal demise were determined by manual review of the EHR (GJDH). When more than one condition (maternal, fetal, placental, umbilical, etc.) was present, the primary or most serious complication was the one counted as cause for this study, recognizing that many demises were multifactorial. Attribution of CMV to demise was based on CMV infection confirmed in fetal or placental tissue or autopsy specimens using polymerase chain reaction (PCR), viral culture, or immunohistochemical staining, which are standard for establishing congenital infection within the first 21 days of life [11]. Cases were classified as attributable only when CMV DNA was detected in fetal or placental tissue by PCR, culture, or immunohistochemistry, and the clinical phenotype was consistent with congenital CMV. Cases lacking tissue confirmation but with maternal CMV findings suggestive of active infection during pregnancy (e.g., positive IgM and/or low-avidity IgG when available), prenatal imaging findings compatible with congenital CMV (including fetal growth restriction, hepatosplenomegaly, ascites, microcephaly, ventriculomegaly, intracranial calcifications, or other central nervous system abnormalities), and no definitive fetal or placental tissue confirmation were classified as “possible” [9,12]. Cases with positive serology but negative testing for CMV of fetal or placental tissue were classified as not attributable to CMV. When available in the record, stillbirth attributable to other etiologies, infectious and non-infectious, was collected.

Maternal data included age, parity, and CMV testing results (IgG, IgM, avidity, and DNA from amniotic fluid when available) [13], with a positive IgM being used as evidence of active or recent CMV infection. Fetal and neonatal variables encompassed gestational age at demise, CMV-specific testing, and placental pathology [14]. Fetal imaging reports were reviewed for evidence of fetal growth restriction, hepatosplenomegaly, ascites, microcephaly, ventriculomegaly, intracranial calcifications, or other central nervous system malformations [15].

Data abstraction involved automated EHR queries supplemented by manual chart review to validate CMV positivity and clinical context. Descriptive statistics summarized demographic and clinical characteristics. Due to sparse cell counts, Fisher’s exact testing was used rather than chi-square analysis.

This study, as well as data collection and analysis for this project, was approved by the Institutional Review Board of Baylor College of Medicine and Affiliated Institutions approved (Protocol: H-55386) “REFACE cCMV: A Real-World Feasibility Study To Examine The Epidemiology Of Congenital Cytomegalovirus (CMV) Infection In Texas, USA” As this was a retrospective chart review, consent was waived by the Baylor College of Medicine and Affiliated Institutions Institutional Review Board.

## 3. Results

**Figure 1:**
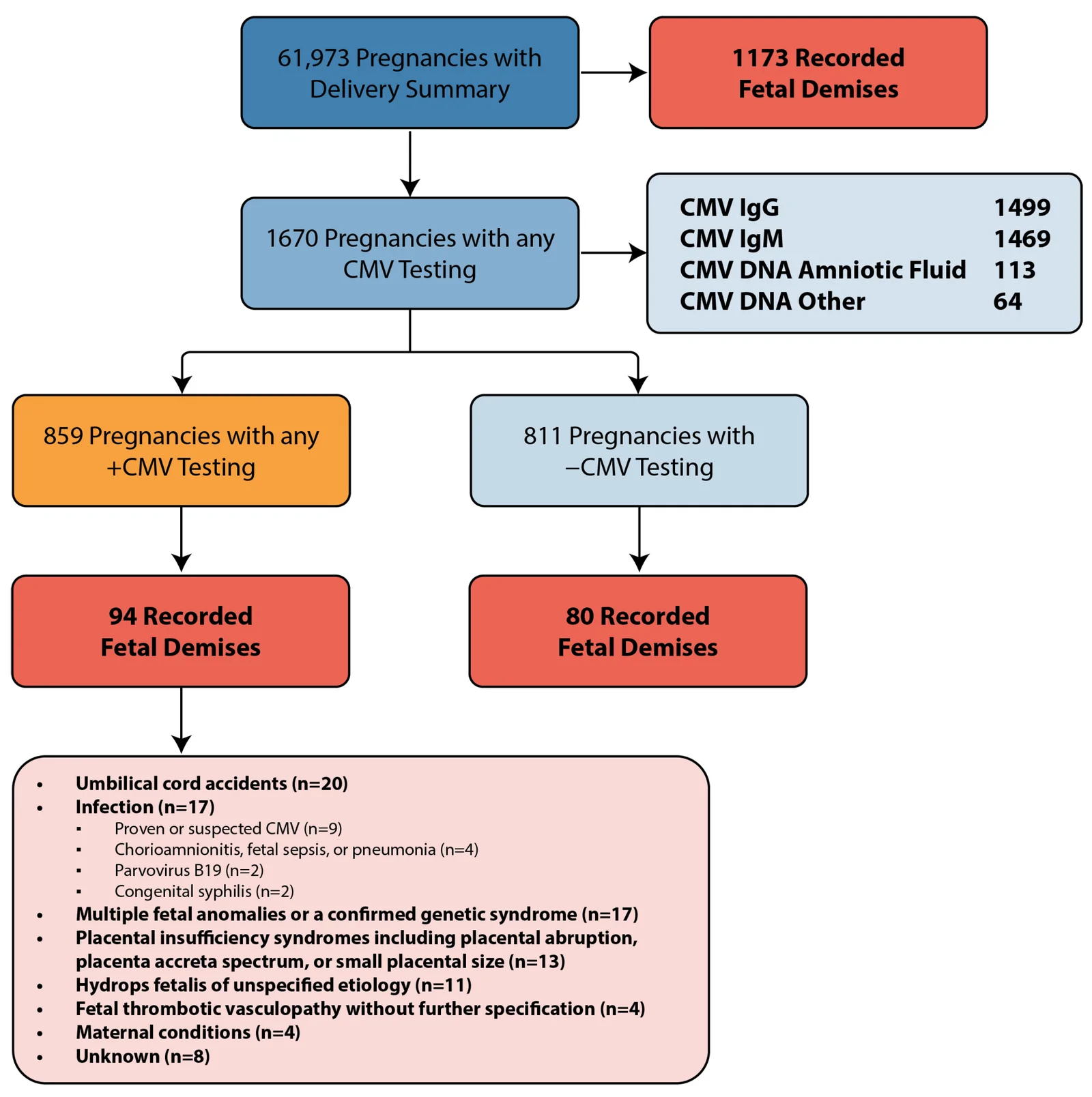
Flow diagram for identification of cases for this retrospective study. Of 61,973 pregnancies in our system for which we had a delivery summary, 1670 had any testing for CMV recorded within our EHR. Of these pregnancies, 859 had any positive test for CMV (e.g., CMV IgG, CMV IgM, etc.), in which there were 94 recorded fetal demises. CMV DNA Other (i.e., DNA detection other than in Amniotic Fluid).

In the study period, there were 61,973 pregnancies in our system for which we had delivery summaries, of which 1173 resulted in fetal demise (Figure 1). Of the nearly 62,000 pregnancies, 1670 had any CMV testing done, 859 with abnormal/positive testing (96 demises), and 811 with normal/negative testing (80 demises). Of the 96 pregnancies that resulted in infant demise in which positive maternal CMV testing was documented during the pregnancy, there were 2 in which we had no available fetal testing. Of the 94 demises in which we had maternal and fetal testing available, there were 4 cases (4.3%) attributable to CMV. Of these 94 demises, 25 (27%) occurred in primiparous individuals, with a majority occurring in the second and third trimesters, with a mean gestational age of 28.4 weeks ± 6.6. In the cases where maternal CMV testing was consistent with active, recent infection in pregnancy (i.e., a positive IgM), the mean gestational age was 23 weeks, and for those with demise attributable to CMV, the mean gestational age at demise was 23.7 weeks ± 3.7. Cohort demographics are listed in Supplementary Table S1.

**Figure 2:**
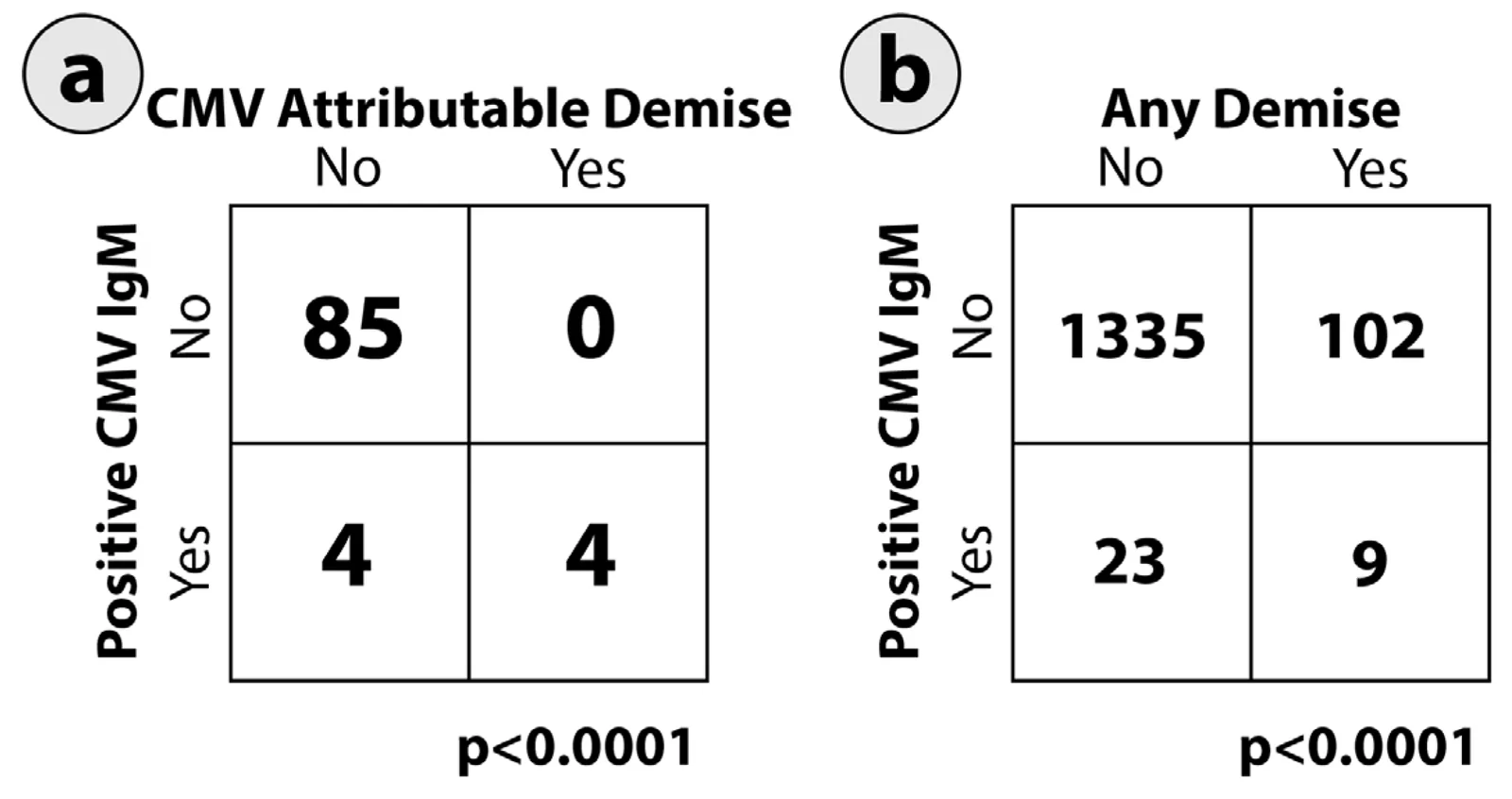
Association between maternal CMV IgM during pregnancy and fetal demise. Fisher’s exact testing of the proportions of demise attributable to CMV when an IgM was present in the mother was significant (**a**). Of all pregnancies in which a CMV IgM was performed (*n* = 1469), a positive CMV IgM was more common in pregnancies that resulted in fetal demise (**b**).

A detailed breakdown of maternal testing is shown in Supplementary Table S2. Given how we identified cases, all but one pregnancy had a result for both CMV IgM/IgG (amniocentesis positivity by PCR in amniotic fluid identified this case). A CMV IgM was performed in 93 of the 94 cases, with 9 positives (9.7%). CMV DNA detection from amniocentesis was performed in 10 of the 94 cases, with 2 positive (20%). Of the 94 cases, CMV testing of fetal tissue was performed 37 times with 3 positive results (8.1%), and CMV testing of placental tissue was performed 85 times with 3 positive results (3.5%). There were no pregnancies with demise attributable to CMV in which a positive CMV IgM was not present (Figure 2). Of the 93 pregnancies in which both IgM and IgG serologic testing for CMV was performed, 9 (9.7%) demonstrated serologic evidence of active infection (Table 1) [16]. Serologic evidence of active CMV infection was significantly associated with fetal demise (*p* < 0.001) (Figure 2) [17]. Serologic evidence of active CMV infection during pregnancy was more common among pregnancies resulting in demise than among those without demise, suggesting that maternal CMV reactivation or primary infection may mark increased vulnerability. Clinical, diagnostic, and pathologic characteristics of stillbirths occurring in pregnancies with a positive CMV IgM recorded during pregnancy (Table 1) are presented in Supplementary Table S3.

Among the 94 fetal demises reviewed, causes included chorioamnionitis, fetal sepsis, or pneumonia (*n* = 4), proven or suspected CMV (*n* = 9), parvovirus B19 (*n* = 2), congenital syphilis (*n* = 2), multiple fetal anomalies or a confirmed genetic syndrome (*n* = 17), hydrops fetalis of unspecified etiology (*n* = 11), umbilical cord accidents (*n* = 20), placental insufficiency syndromes including placental abruption, placenta accreta spectrum, or small placental size (*n* = 13), fetal thrombotic vasculopathy without further specification (*n* = 4), and maternal conditions (*n* = 4). The cause of demise remained unknown in 8 cases.

## 4. Discussion

This study provides further evidence that congenital CMV infection is an identifiable cause in a subset of fetal demise cases. While CMV is a recognized cause of fetal demise, this study demonstrates that application of stringent virologic confirmation criteria can distinguish maternal CMV exposure from confirmed CMV-attributable fetal death. Even under these conservative criteria, CMV remained an identifiable contributor to fetal demise, supporting continued consideration of CMV in the evaluation of otherwise unexplained stillbirth.

Our findings align with existing literature describing the spectrum of congenital CMV disease, which ranges from asymptomatic infection to severe multisystem involvement [18], and underscores the virus’s capacity for widespread tissue injury [6]. In contrast to the 7–15% CMV detection rates reported in systematic reviews of stillbirth pathology [7,9,19,20], 4.3% of CMV-positive pregnancies in our cohort met strict criteria for etiologic attribution. This discrepancy highlights the distinction between viral detection and causal inference and underscores the importance of standardized postmortem testing protocols. Because attribution required direct evidence of congenital infection and compatible clinical findings, our estimates should be interpreted as a measure of confirmed CMV-attributable fetal demise rather than the broader prevalence of CMV exposure, maternal seropositivity, or CMV detection among stillbirths.

The pathogenesis of CMV-related demise likely reflects both direct viral cytopathic effects and immune-mediated damage [21]. Viral replication in the placenta can impair nutrient exchange and oxygen delivery, while infection of neural progenitor cells disrupts brain development, resulting in microcephaly and cortical malformations [22]. While mechanistic evaluation was beyond the scope of this study, direct virologic confirmation of CMV in fetal or placental specimens supports the biologic plausibility of the pathogenic pathways described above and their potential contribution to fetal demise. While these mechanisms mirror those described in other congenital infections, such as Zika virus, it should be noted that CMV remains far more prevalent worldwide.

Our findings are important both clinically as well as in consideration of public health policy. Despite the high burden of disease, routine maternal screening for CMV is not standard practice in the United States, and universal newborn screening remains limited to a few states [23]. Current U.S. stillbirth evaluation guidelines emphasize syphilis testing but do not recommend routine CMV testing, despite accumulating evidence that CMV is frequently detected in placental and fetal tissues [24,25,26]. Legislative efforts in recent years have expanded targeted testing for infants who fail hearing screens, but this approach misses many cases, particularly those resulting in fetal demise [27].

Preventive strategies, including maternal antiviral therapy and hyperimmune globulin administration, have produced mixed results across studies. Although large randomized controlled trials did not demonstrate a significant reduction in congenital CMV infection with hyperimmune globulin therapy [28,29], observational studies and long-term follow-up cohorts have reported potential benefits when treatment was administered early after primary maternal infection and according to specific dosing regimens [30,31]. Similarly, while valacyclovir has shown promise in some studies of primary maternal infection [32,33], additional evidence is needed to better define its role in clinical practice [34]. Ultimately, development of a safe and effective CMV vaccine remains the most compelling long-term solution, yet decades of research have not yielded a licensed product [35]. Education campaigns promoting preventive behavioral practices among pregnant individuals, such as handwashing after contact with young children and avoiding contact with saliva and other body secretions from young children, are the only effective prevention strategy so far, but remain underutilized [22].

A major contribution of this study is the distinction between maternal CMV exposure, maternal CMV serologic findings, and confirmed CMV-attributable fetal demise. Although maternal CMV IgM positivity was identified in nine pregnancies resulting in fetal demise, only four met stringent criteria for confirmed attribution. This finding underscores the limitations of relying on maternal serology alone and highlights the value of fetal and placental virologic testing when CMV is suspected. While our findings do not support attributing fetal demise to CMV solely on the basis of maternal serology, they do suggest that standardized evaluation of fetal and placental tissues may improve identification of confirmed CMV-associated fetal deaths.

Strengths of this study include the use of direct virologic confirmation and a relatively large cohort of fetal demises. Limitations include its retrospective design, incomplete ascertainment of maternal infection timing, single-center setting, and potential referral bias. These factors, along with case ascertainment restricted to virologically confirmed infections, may contribute to the lower proportion of demises attributable to CMV observed in our study (4.3%) compared with prior estimates ranging from 7 to 15%, and may limit the generalizability of our findings to other populations. Furthermore, because this study was conducted at a large tertiary/quaternary referral center specializing in maternal-fetal medicine and fetal care, the underlying prevalence of fetal demise in our cohort exceeds that reported in population-based studies. Referral bias toward medically complex pregnancies should therefore be considered when interpreting these findings and their generalizability. Future research should focus on prospective surveillance, integration of universal screening strategies, and evaluation of antiviral interventions in high-risk pregnancies while continuing to rely on direct virologic confirmation of CMV.

## 5. Conclusions

Confirmed congenital CMV infection accounts for a substantial proportion of fetal demises. These findings highlight the value of standardized screening protocols, preventive strategies, and public health education to mitigate the impact of CMV on perinatal outcomes. As legislative momentum grows and vaccine development advances, addressing CMV should remain a priority for both clinicians and policymakers.

## Figures and Tables

**Table 1:** Characteristics of infants born stillborn to mothers with a positive CMV IgM recorded during pregnancy. Prenatal count is the number of encounters recorded within our system during the selected pregnancy. This table includes all fetal demise pregnancies with maternal CMV IgM positivity and should not be interpreted as a case series of CMV-attributable fetal demise.

Characteristics of Pregnancies Resulting in Stillbirth in Mothers with a Positive CMV IgM Recorded During Pregnancy												
Pre-Pregnancy Maternal Age (Years)	GP Count	Stillbirth Attributable to CMV	Maternal CMV Serology	CMV Avidity	CMV DNA in Amniotic Fluid	CMV DNA from Tissue Fetal/Neonatal	CMV DNA from Placental Tissue	Prenatal Encounter Count *	Delivery Gestational Age (Weeks/Days)	Language	Patient Ethnic Group	Patient Race
19	G3P1	Yes	CMV IgG+, CMV IgM+	Not Done	Not Done	Positive	Positive	1	27 w 6 d	English	Not Hispanic or Not Latino	Black or African American
33	G3P1	Yes	CMV IgG+, CMV IgM+	Not Done	Positive	Positive	Positive	0	21 w 0 d	English	Hispanic or Latino	White
24	G1P0	Yes	CMV IgG+, CMV IgM+	Not Done	Not Done	Positive	Positive	6	22 w 1 d	English	Not Hispanic or Not Latino	Asian
33	G2P1	Yes	CMV IgG+, CMV IgM+	High Avidity	Positive	Not Done	Not Done	4	30 w 0 d	English	Not Hispanic or Not Latino	White
23	G3P2	Possible	CMV IgG+, CMV IgM+	Low Avidity	Not Done	Not Done	Not Done	6	28 w 1 d	English	Not Hispanic or Not Latino	White
37	G5P2	No	CMV IgG+, CMV IgM+	High Avidity	Not Done	Negative	Negative	3	22 w 5 d	English	Not Hispanic or Not Latino	White
23	G2P0	No	CMV IgG+, CMV IgM+	Not Done	Not Done	Negative	Negative	0	13 w 0 d	English	Not Hispanic or Not Latino	White
35	G2P1	No	CMV IgG+, CMV IgM+	Not Done	Negative	Negative	Negative	0	27 w 6 d	English	Not Hispanic or Not Latino	White
30	G2P0	No	CMV IgG+, CMV IgM+	Not Done	Not Done	Not Done	Negative	2	19 w 5 d	English	Not Hispanic or Not Latino	White

Abbreviations: CMV, cytomegalovirus; GP, gravida-para; IgM, immunoglobulin M. Note: * Prenatal count is the number of encounters recorded within our system during the selected pregnancy.

## Data Availability

Data cannot be shared publicly because of the vulnerable nature of the patients included in this study (children, cognitive impairment), sharing of a full de-identified data set for the purposes of publication is limited by the Institutional Review Board of Baylor College of Medicine and Affiliated Institutions (Protocol: H-55386) which states “when analyzed and presented or published, will be presented in aggregate data only”. As such, data within this manuscript can be made available upon request, and the decision to make available is not restricted by the funder/sponsor. Data are available from the Baylor College of Medicine Institutional Review Board of Baylor College of Medicine and Affiliated Institutions for researchers who meet the criteria for access to confidential data through the following contact information: irb@bcm.edu, phone +1-713-798-6970. Requests for data should be made to either the corresponding author or IRB (as above).

## Supplementary Materials

The following supporting information can be downloaded at: https://www.mdpi.com/article/10.3390/v18080881/s1, Table S1: Demographics for mothers with a history of fetal demise who had positive CMV testing during the affected pregnancy. Table S2: Results of CMV testing performed in stillbirths in which any CMV test was abnormal. All individuals had a positive IgG, and there was no isolated IgM elevation in the absence of IgG elevation. CMV DNA detection through amniocentesis was not common, and while examination of fetal tissue for CMV only occurred in 39% of cases, nearly 90% of placentas were examined for evidence of CMV. Table S3: Clinical, diagnostic, and pathologic characteristics of stillbirths occurring in pregnancies with a positive CMV IgM recorded during pregnancy. Cases were classified as proven congenital CMV when CMV infection was confirmed by amniotic fluid PCR, placental histopathology demonstrating CMV inclusions, fetal tissue pathology demonstrating CMV inclusions, or a combination of these findings. Cases were classified as possible congenital CMV when maternal serologic evidence of infection and/or fetal findings consistent with congenital CMV were present but direct virologic or pathologic confirmation was lacking. Cases with positive serology but negative testing of fetal or placental tissue were classified as not being attributable to CMV. Gestational age (GA) indicates estimated gestational age at fetal demise or delivery. Major fetal findings include prenatal ultrasound abnormalities and autopsy findings when available. Alternative etiologies refer to competing explanations for fetal demise identified during clinical, genetic, placental, or pathologic evaluation. Abbreviations: CMV, cytomegalovirus; GA, gestational age; IUGR, intrauterine growth restriction; IUFD, intrauterine fetal demise; PCR, polymerase chain reaction; PMG, polymicrogyria.
