# Supplementary material for: Congenital Cytomegalovirus Infection in Fetal Demise: A Retrospective Cohort Study
Source: Viruses. 2026 Aug 12;18(8):881. doi: 10.3390/v18080881 (PMC13517689; doi:10.3390/v18080881)
Supplement: Supplementary file 1 [file viruses-18-00881-s001.zip › viruses-4389310-supplementary.pdf]

Marital Status	Count	Percent
Divorced	2	2%
Married	49	52%
Other	1	1%
Separated	2	2%
Significant Other	8	9%
Single	32	34%
**Language**	**Count**	**Percent**
Arabic	1	1%
English	87	92%
Spanish	7	7%
**Ethnicity**	**Count**	**Percent**
Hispanic or Latino	34	36%
Not Hispanic or Latino	59	62%
Unable to Obtain	1	1%
**Race**	**Count**	**Percent**
Asian	7	7%
Black or African American	34	36%
Other	1	1%
Unable to Obtain	1	1%
White	51	54%

Supplementary Table S1: Demographics for mothers with a history of fetal demise who had positive CMV testing during the affected pregnancy.

CMV IgG	Count	Percent
Positive	94	100%
**CMV IgM**	**Count**	**Percent**
Negative	84	89%
Positive	9	10%
Not Done	1	1%
**CMV IgG Avidity**	**Count**	**Percent**
Negative	0	0%
Positive	13	14%
Low Avidity	1	(8%)
Intermediate Avidity	1	(8%)
High Avidity	11	(84%)
Not Done	81	86%
**CMV DNA Amniocentesis**	**Count**	**Percent**
Negative	8	9%
Positive	2	2%
Not Done	84	89%
**CMV in Fetal Tissue**	**Count**	**Percent**
Negative	34	36%
Positive	3	3%
Not Done	57	61%
**CMV in Placenta**	**Count**	**Percent**
Negative	81	87%
Positive	3	3%
Not Done	9	10%

Supplementary Table S2: Results of CMV testing performed in stillbirths in which any CMV test was abnormal. All individuals had a positive IgG, and there was no isolated IgM elevation in the absence of IgG elevation. CMV DNA detection through amniocentesis was not common, and while examination of fetal tissue for CMV only occurred in 39% of cases, nearly 90% of placentas were examined for evidence of CMV.

Stillbirth Attributable to CMV	GA at Demise	Maternal CMV Testing	Evidence of Congenital CMV	Major Fetal Findings	Placental Findings	Alternative Etiology	Other Studies
No	27 wk	IgM+, IgG+	Amnio PCR negative; placenta PCR negative; autopsy negative	None reported	Negative for CMV by PCR	Possible thrombophilia	RPR non-reactive, Parvovirus IgG and IgM negative
Yes	27 wk	IgM+, IgG+	Placenta CMV+, fetal tissues CMV+	Ventriculomegaly, echogenic bowel, microcephaly, brain calcifications, PMG	Diffuse lymphoplasmacytic villitis, fetal vasculopathy, positive for CMV by PCR	None identified	RPR Nonreactive, HIV negative, HSV ½ IgG positive, toxoplasmosis IgG and IgM negative, VZV IgG positive, Chlamydia/GC urine screen negative
No	19 wk	IgM+, IgG+	No fetal pathology performed	Omphalocele	Basal plate separation, acute choriodeciduitis	IVF, incompetent cervix, chorioamnionitis	Gonorrhea/chlamydia urine screen negative, VZV IgG positive, Rubella IgG positive, HIV negative, RPR non-reactive
Yes	21 wk	IgM+, IgG+	Amnio PCR+, placenta CMV+, fetal inclusions	IUGR, hydrops, ascites, echogenic bowel, cerebral calcifications, cardiomegaly	Villitis, lymphoplasmacytic inflammation, CMV inclusions, positive for CMV by PCR	None identified	RPR Nonreactive, Toxoplasmosis IgG and IgM negative, HSV ½ IgG negative, VZV IgG positive, Rubella IgG positive and IgM negative, Parvovirus IgG positive and IgM negative, Zika virus PCR negative, Coxsackie IgM/IgG negative
No	22 wk	IgM+, IgG+, high avidity	Placenta PCR negative; autopsy negative	Twin gestation	Abruption	Placental abruption	RPR Nonreactive
Possible	27 wk	IgM+, IgG+, low avidity	Inflammation only, no placental or fetal studies found in record	IUGR, hydrops, ascites, pleural/pericardial effusions, echogenic bowel	Inflammation only	None identified	Toxoplasmosis IgG and IgM negative, Rubella IgG positive, Gonorrhea/Chlamydia urine screen negative, HIV negative, RPR Nonreactive
Yes	22 wk	IgM+, IgG+	Placental CMV inclusions; fetal CMV inclusions	Echogenic bowel; multisystem CMV disease	Villitis/deciduitis with CMV inclusions	Mixed bacterial growth noted	Rubella IgG positive

No	13 wk	IgM+, IgG+	Placenta PCR negative; autopsy negative	Trisomy 18, cystic hygroma	Negative CMV	Trisomy 18	Toxoplasmosis IgG and IgM negative, Parvovirus IgG positive and IgM negative, Rubella IgG positive, HIV negative, RPR Nonreactive, Gonorrhea/Chlamydia urine screen negative
Yes	30 wk	IgM+, IgG+, high avidity	Amniotic fluid PCR+	IUGR, ventriculomegaly, PMG, echogenic bowel	Not available	None apparent	None

Supplementary Table S3: Clinical, diagnostic, and pathologic characteristics of stillbirths occurring in pregnancies with a positive CMV IgM recorded during pregnancy. Cases were classified as proven congenital CMV when CMV infection was confirmed by amniotic fluid PCR, placental histopathology demonstrating CMV inclusions, fetal tissue pathology demonstrating CMV inclusions, or a combination of these findings. Cases were classified as possible congenital CMV when maternal serologic evidence of infection and/or fetal findings consistent with congenital CMV were present but direct virologic or pathologic confirmation was lacking. Cases with positive serology but negative testing of fetal or placental tissue were classified as not being attributable to CMV. Gestational age (GA) indicates estimated gestational age at fetal demise or delivery. Major fetal findings include prenatal ultrasound abnormalities and autopsy findings when available. Alternative etiologies refer to competing explanations for fetal demise identified during clinical, genetic, placental, or pathologic evaluation. Abbreviations: CMV, cytomegalovirus; GA, gestational age; IUGR, intrauterine growth restriction; IUFD, intrauterine fetal demise; PCR, polymerase chain reaction; PMG, polymicrogyria
